# Ion concentrations in CSF and serum are differentially and precisely regulated

**DOI:** 10.1093/braincomms/fcaf201

**Published:** 2025-05-24

**Authors:** Tim Lyckenvik, My Forsberg, Kalle Johansson, Markus Axelsson, Henrik Zetterberg, Kaj Blennow, Sebastian Illes, Pontus Wasling, Eric Hanse

**Affiliations:** Department of Physiology, Institute of Neuroscience and Physiology, Sahlgrenska Academy at the University of Gothenburg, SE-405 30 Gothenburg, Sweden; Department of Neurology, Sahlgrenska University Hospital, SE-413 45 Gothenburg, Sweden; Department of Physiology, Institute of Neuroscience and Physiology, Sahlgrenska Academy at the University of Gothenburg, SE-405 30 Gothenburg, Sweden; Department of Clinical Neuroscience, Institute of Neuroscience and Physiology, Sahlgrenska Academy at the University of Gothenburg, SE-413 45 Gothenburg, Sweden; Department of Neurology, Sahlgrenska University Hospital, SE-413 45 Gothenburg, Sweden; Department of Clinical Neuroscience, Institute of Neuroscience and Physiology, Sahlgrenska Academy at the University of Gothenburg, SE-413 45 Gothenburg, Sweden; Department of Psychiatry and Neurochemistry, Institute of Neuroscience and Physiology, Sahlgrenska Academy at the University of Gothenburg, SE-413 45 Gothenburg, Sweden; Wisconsin Alzheimer’s Disease Research Center, School of Medicine and Public Health, University of Wisconsin-Madison, Madison, WI 53792, USA; Clinical Neurochemistry Laboratory, Sahlgrenska University Hospital, SE-431 41 Mölndal, Sweden; Department of Neurodegenerative Disease, UCL Institute of Neurology, London WC1N 3BG, UK; UK Dementia Research Institute at UCL, London NW1 3BT, UK; Hong Kong Center for Neurodegenerative Diseases, Clear Water Bay, Hong Kong, China; Department of Psychiatry and Neurochemistry, Institute of Neuroscience and Physiology, Sahlgrenska Academy at the University of Gothenburg, SE-413 45 Gothenburg, Sweden; Clinical Neurochemistry Laboratory, Sahlgrenska University Hospital, SE-431 41 Mölndal, Sweden; Paris Brain Institute, ICM, Pitié-Salpêtrière Hospital, Sorbonne University, FR-75013 Paris, France; Neurodegenerative Disorder Research Center, Division of Life Sciences and Medicine, and Department of Neurology, Institute on Aging and Brain Disorders, University of Science and Technology of China and First Affiliated Hospital of USTC, Hefei 230001, P.R. China; Department of Physiology, Institute of Neuroscience and Physiology, Sahlgrenska Academy at the University of Gothenburg, SE-405 30 Gothenburg, Sweden; Department of Neurology, Sahlgrenska University Hospital, SE-413 45 Gothenburg, Sweden; Department of Clinical Neuroscience, Institute of Neuroscience and Physiology, Sahlgrenska Academy at the University of Gothenburg, SE-413 45 Gothenburg, Sweden; Department of Physiology, Institute of Neuroscience and Physiology, Sahlgrenska Academy at the University of Gothenburg, SE-405 30 Gothenburg, Sweden

**Keywords:** CSF, blood–brain barrier, potassium, chloride, magnesium

## Abstract

Fluctuations of extracellular brain ion concentrations have been associated with transitions between brain states such as sleep and wakefulness, and disturbances have been implicated in a variety of neurological conditions, including dementia, epilepsy and migraine. This study aims to define the normal CSF ion profile and identify key factors influencing its regulation. In this cross-sectional study, we analysed samples from 42 individuals (16 men), including 28 healthy participants and 14 patients with medically unexplained neurological symptoms. Age spanned between 20 and 55 years. Using validated clinical assays, we measured paired CSF and serum concentrations of Ca²⁺, Cl⁻, K⁺, Mg²⁺ and Na⁺. We examined their interrelationships and assessed the impact of blood–brain barrier permeability (the albumin quotient), age, sampling time and sex. CSF ion concentrations were highly stable and maintained within distinct, narrow ranges, separate from serum ranges for all ions measured (*P* < 0.0001), with no overlapping. Cl^−^ (+25%), Na^+^ (+5%) and Mg^2+^ (+37%) concentrations were higher, while K^+^ (−30%) and Ca^2+^ (−50%) concentrations were lower. Consistent with an independent and active CNS homeostatic control, CSF concentrations of K^+^, Cl^−^ or Mg^2+^ showed no significant correlation with their serum counterparts, while Na^+^ and Ca^2+^ displayed moderate associations with serum levels. Moreover, blood–brain barrier permeability had no significant impact on CSF ion concentrations. Small, but significant, age-related declines were observed for Cl^−^, Mg^2+^ and Ca^2+^ in CSF, and circadian fluctuations affected K^+^, which increased slightly in the afternoon. Minor sex differences were noted, with men exhibiting slightly higher Mg^2+^ and Ca^2+^ levels. Our findings demonstrate that CSF ion concentrations are precisely regulated at the barriers of the CNS, largely independent of serum levels and with low interindividual variation, reinforcing the concept of a highly controlled CNS environment. This distinct ion composition may help modulate neuronal excitability, supporting brain state transitions such as sleep–wake cycling. Identification of hydration status as a potential confounding factor suggests that normalization to CSF-Na^+^ may improve the detection of pathological disruptions in CSF ion homeostasis. These insights reinforce the foundation for using CSF ion profiles as biomarkers for neurological disorders.

## Teaser

Lyckenvik *et al.* show that CSF ion concentrations are precisely regulated, distinct from serum and unaffected by blood–brain barrier integrity. Their findings provide normative data from healthy individuals and highlight CNS-specific mechanisms for ion homeostasis, offering insights into CSF ions’ potential roles in physiological and pathological neuronal excitability.

## Graphical Abstract

**Graphical Abstract:**
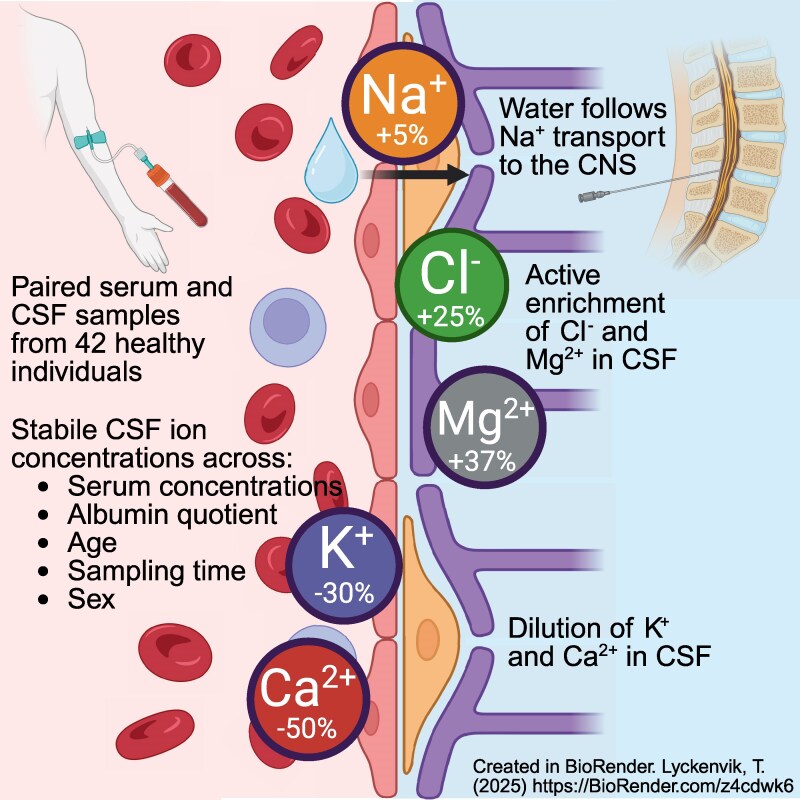

## Introduction

Emerging research suggests that the brain’s extracellular ion composition potentially governs transitions between brain states, such as wakefulness and sleep, by modulating neuronal activity. The ion composition of the CSF is actively regulated and not merely an ultrafiltrate of serum.^1,2^ Notably, CSF concentrations of Ca^2+^,^3^ Mg^2+^,^4-8^ and K^+9-14^ remain remarkably stable despite significant fluctuations in their serum concentrations.

Brain extracellular K^+^ concentrations decrease in association to sleep in mice and increase when the mice return to wakefulness, while extracellular Ca^2+^ and Mg^2+^ concentrations appear to change inversely.^15^ Experimental manipulation of brain extracellular ion composition accordingly,^15^ and lowering K^+^ concentrations in isolation,^16^ demonstrated the ability to switch brain states between wakefulness and sleep in the mice. In line with these findings, our previous work gave support to the hypothesis that brain ion composition is dynamically regulated in synchrony with different brain states,^17^ by showing that CSF K^+^ concentrations in humans are lower in the early morning compared to the afternoon, consistent with a circadian regulation.^18^

Changes in extracellular K^+^ levels can directly influence behaviour, as shown in studies on awake, free moving mice. Elevated extracellular K^+^ is linked to heightened alertness, locomotion and motor performance.^16,19^ Moreover, extracellular K^+^ and stimulatory neuromodulators appear to modulate each other independently of synaptic activity. Specifically, stimulatory neuromodulators can elevate extracellular K^+^ even when action potentials are blocked,^15^ and small experimental increases in K^+^ levels can increase interstitial fluid (ISF) concentrations of dopamine, norepinephrine and serotonin.^16^

CSF sampling offers a window into the composition of brain ISF, due to the free communication between these fluids^20,21^ resulting in negligible differences in K^+^ concentrations between CSF and ISF.^11^ This methodological advantage allows studying changes in CNS extracellular ion composition which have been linked to various conditions, including dementia with Lewy bodies,^22^ epilepsy,^23^ major depression^24^ and migraine.^25,26^ Likewise, genetic alterations in astrocytic regulation of brain K^+^ concentrations have been implicated in schizophrenia,^27^ epilepsy^28^ and Huntington’s disease,^29^ and experimental evidence highlights the role of ISF ion dynamics in brain oedema formation.^30,31^

Despite these implications, CSF ion concentrations are rarely reported in clinical studies, potentially because our understanding of physiological brain ion homeostasis remains limited. Therefore, in this exploratory study, we analyse CSF samples from young to middle-aged healthy individuals quantifying five key ions expected to impact neuronal excitability: Ca^2+^, Cl^−^, K^+^, Mg^2+^ and Na^+^. We also examine their relationships with paired serum concentrations, blood–brain barrier (BBB) permeability, age, sample collection timing and sex. We hypothesized that these ions are tightly regulated within narrow physiological ranges, largely independent of serum levels, but potentially modulated by these other factors. This work aims to establish foundational data for future investigations of normal and pathological fluctuations in CNS ion homeostasis.

## Materials and methods

### Study design and participants

This non-interventional, cross-sectional, retrospective study was conducted at the outpatient clinic of the Department of Neurology, Sahlgrenska University Hospital in Gothenburg, Sweden. A complete diagnostic evaluation was carried out by an experienced neurologist.

**Figure 1:**
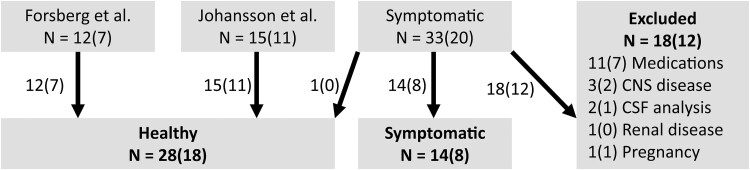
**Flow chart of inclusion and exclusion from three analysis batches.** Total number (number of females). Forsberg *et al*., participants originally recruited for Forsberg *et al*.^18^; Johansson *et al*., participants originally recruited for Johansson *et al*.^32^; Symptomatic, 1 additional healthy volunteer and 32 patients referred to specialist neurologic investigation due to neurological symptoms, but who had no confirmed neurological diagnosis at the time of their retrospective identification; Healthy and Symptomatic, defined subgroups.

A total of 42 subjects were included in the study. They were divided into two groups: healthy subjects and symptomatic subjects (Fig. 1). The healthy subjects included 28 participants recruited from the local community.^18,32^ Prior to inclusion, all subjects in the healthy group underwent screening for the exclusion criteria of symptoms or diagnosis potentially related to the CNS, intake of medications or drugs and abnormal sleep habits or general health.

The symptomatic subjects consisted of 14 patients who were referred to the Department of Neurology at the Sahlgrenska University Hospital in Gothenburg, Sweden, due to neurological symptoms, but had no confirmed neurological diagnosis after diagnostic workup at the time of their retrospective identification. Originally, 32 subjects were identified in November 2022 by searching our biobank for diagnosis codes Z03 (encounter for medical observation for suspected diseases and conditions ruled out), R20.1 (hypoesthesia of skin), R20.2 (paraesthesia of skin) and R29.8 (other symptoms and signs involving the nervous and musculoskeletal systems) according to the International Statistical Classification of Diseases and Related Health Problems, 10th edition (ICD-10). Their medical records were screened to identify concurring conditions or medications assumed to have potential influence on CSF or serum ion homeostasis, resulting in the exclusion of 16 subjects. In this stage, data from three participants were excluded due to suspected misdiagnosis and 13 subjects were excluded due to the presence of concurrent conditions (untreated gout, pregnancy) or medications potentially affecting CSF (carbamazepine, gabapentin, clonazepam, bupropion, clomipramine, sertraline, venlafaxine, lithium, risperidone, tramadol or propofol plus midazolam during the lumbar puncture) or serum (amlodipine, enalapril, losartan, magnesium, metoprolol, metformin and vitamin D) electrolyte composition. Comorbidities such as anaemia, anxiety with depression, fibromyalgia, a foramen Monroi cyst, lumbar disc herniation without medullary compression and irritable bowel syndrome (found in one subject each) were not grounds for exclusion. Data from another two subjects in this group were later excluded due to abnormal CSF baseline biomarkers, resulting in a total of 14 symptomatic subjects remaining.

### Ethical approval and consent

The study was approved by the Swedish Ethical Review Authority in Gothenburg (protocol numbers: #223-15, #460-13, #492-18 and #942-12). All participants provided oral and written informed consent prior to inclusion. The study was conducted in accordance with the Declaration of Helsinki.

### CSF analysis

CSF samples were obtained by routine lumbar puncture, performed by an experienced neurologist using an atraumatic needle (22-25G). After collection, the CSF samples were centrifuged at 2000 g for 10 min at room temperature to remove cells and debris. The supernatant was aliquoted and stored at −80°C pending biochemical analysis.

The samples were analysed in three batches:

1. Serum (albumin and osmolality only) and CSF samples from 12 healthy subjects (7 females, 5 males) were collected and analysed between January and September 2019, as part of Forsberg *et al*.^18^ Three separate samples [afternoon (3–5 p.m.), morning after sleep deprivation and morning after sleep (6–7 a.m.)] from each subject were sampled in randomized order, separated by at least 4 weeks. Originally, only the afternoon samples were included in this analysis. However, to account for potential batch effects, morning after sleep samples were added in a separate correlation analysis on age and sampling timing effects.
2. Serum and CSF samples from 15 additional healthy subjects (11 females, 4 males) were collected as part of Johansson *et al*.,^32^ between February and August 2018, within ±1.5 h of noon. Serum from one (female) individual was missing.
3. Serum and CSF samples from 32 symptomatic patients (of which 14 were finally included in the study) were collected between June 2013 and February 2017 as part of their diagnostic evaluation, including a diagnostic lumbar puncture performed sometime between 9 a.m. and 2:30 p.m. during which an extra 10 ml CSF and blood, respectively, were stored for later research purposes. One additional CSF sample from a healthy male subject was collected in December 2022 and included in this analysis batch.

Two symptomatic patients were excluded due to slightly abnormal CSF findings [elevated CSF leukocyte count or oligoclonal IgG bands (OCBs) present selectively in CSF]. All remaining subjects had albumin quotient (Q_alb_) within the age-normalized reference interval, CSF leukocyte count lower than 3 × 10^6^/l and four or fewer OCBs selectively in CSF (two subjects had three to four OCBs, one had two and one had one). Healthy CSF was not screened for leukocyte count or OCBs.

All measurements were conducted by board-certified laboratory technicians at the Clinical Chemistry Laboratory at Sahlgrenska University Hospital using accredited methods with inter-assay coefficients of variation (CVs) below 2%. The laboratory is accredited under the Swedish Accreditation body (SWEDAC).

Concentrations of Na^+^, K^+^ and Cl^−^ were measured using ion-selective electrodes (ISEs), integrated into the Cobas c 501 instrument (Roche Diagnostics), which are approved for clinical use without clinically relevant interferences. Calibration of the ISE methods were performed at least once daily using ISE Standards Low (S1) and High (S2) (Roche Diagnostics). Ca^2+^ and Mg^2+^ concentrations were determined colorimetrically using the o-cresolphthalein and chlorophonazo III methods, respectively, on the Cobas c 501 instrument (Roche Diagnostics), according to instructions from the manufacturer. The methods were calibrated after each reagent lot change, using Standards Low (S1) and High (S2) (Roche Diagnostics) for Ca^2+^ and Mg^2+^, respectively. Originally, blood ion concentrations were analysed in plasma, but the plasma was collected tubes which did not allow for reliable analyses. Therefore, we chose to analyse serum concentrations instead.

CSF and serum albumin concentrations were measured via immunonephelometry using a Beckman Image Immunochemistry system (Beckman Instruments, Beckman Coulter, Brea, CA, USA). Q_alb_ was calculated as CSF albumin (mg/l) divided by serum albumin (g/l), and only the quotient was reported from the lab. Osmolality was measured as previously described.^33^

Platelet-derived growth factor receptor β (PDGFRβ) was quantified using the Human Total PDGFRβ DuoSet IC ELISA (R&D Systems Europe, Abingdon, UK) with slight adaptations as previously described.^34^ Neurofilament light chain (NfL)^35^ and glial fibrillary acidic protein GFAP^36^ were measured using validated in-house ELISA methodology while t-tau was measured using commercially available Lumipulse technology (Fujirebio, Ghent, Belgium), as previously described.^37^

### Estimation of ionized calcium and magnesium

We estimated the ionized calcium (iCa2^+^_CSF_) by multiplying the total calcium (tCa^2+^) concentrations by our previously^38^ determined ionized proportion. For serum ionized calcium (iCa2^+^_serum_), the fraction of 50% serum iCa^2+^ under normal conditions^39^ was used. A similar approach was applied for Mg^2+^, using reported ionized fractions in serum (69%) and CSF (96%) from a cohort of 10 non-pregnant women,^40^ to estimate ionized fractions (iMg^2+^).

### Statistical analysis

Data were analysed using GraphPad Prism version 10.3.1 for Windows (GraphPad Software, San Diego, CA, USA). Results are reported as median and interquartile range (IQR), unless otherwise specified. Given the limited sample size and the failure of Q_alb_ and age to pass the Shapiro–Wilk normality test, non-parametric tests were employed for analysis. Group differences were assessed using the Mann–Whitney *U* test and paired comparisons of ion concentrations in serum and CSF were analysed using the Wilcoxon test. Correlations between CSF ion concentrations (including the Ca^2+^:Mg^2+^ ratio) and serum concentrations, age, lumbar puncture time and Q_alb_ were examined with Spearman’s rank correlation coefficient. To account for multiple comparisons, *P*-values were adjusted using the Bonferroni method: 5 comparisons for serum–CSF pairs, 24 for correlation analyses and 6 for sex differences. Unadjusted *P*-values are shown in each figure.

For clarity, linear regression was used to fit the best regression line to the scatter plots in the supplementary figures. However, Spearman *r* and associated *P*-values are presented in these figures instead of the *R*^2^ and *P*-values from the linear regression. Fisher’s exact test was used to assess potential sex distribution imbalances between groups. All statistical tests were two-tailed, and statistical significance was set at *P* < 0.05.

## Results

### Demographics

Setting out to characterize the ion composition in CSF of healthy individuals and investigate its regulation, we analysed 28 CSF samples from young to middle-aged healthy subjects. However, 14 paired serum samples from these individuals were unavailable for ion analyses, limiting the power to investigate how CSF ion concentrations are impacted by their serum ion concentrations. We therefore also analysed paired CSF and serum samples from 14 subjects with medically unexplained symptoms by modern standards, for a total of 42 CSF samples.

First, we compared the group of added symptomatic subjects to the group of healthy subjects, to assess if their inclusion could be justified. The sex ratios were comparable (*P* = 0.74); however, the subjects in the symptomatic group were significantly older (median age: 33.0 years versus 24.0 years; *P* = 0.0011) (Table 1). We screened for undetected disease processes in the symptomatic group by analysing levels of the neuronal injury markers NfL and t-tau and the astrocytic activation marker GFAP. The symptomatic group exhibited higher concentrations of NfL (280 pg/ml versus 200 pg/ml; *P* = 0.046) and lower concentrations of GFAP (260 pg/ml versus 378 pg/ml; *P* = 0.026) (Table 1), whereas no significant difference was observed in t-tau levels between the groups (*P* = 0.056). There was no significant sampling time correlation between NfL (*r* = −0.05; *P* = 0.676), GFAP (*r* = 0.23; *P* = 0.15) or t-tau (*r* = 0.07; *P* = 0.68).

**Table 1:** Sex, age, Q_alb_ and CSF parenchymal damage marker distributions between the groups

	All	Healthy	Symptomatic
*N*_CSF_	42	28	14
Male sex, %	16 (38)	10 (36)	6 (43)
Age (years)	28 (23–33)	24 (21–28)	33 (27–39)**
Q_alb_	3.7 (1.3–3.7)	3.6 (2.5–4.3)	3.9 (2.9–4.6)
GFAP (pg/ml)	315 (240–471)	378 (257–507)	260 (173–378)*
NfL (pg/ml)	210 (149–311)	200 (140–260)	280 (188–355)*
t-tau (pg/ml)	230 (187–293)	259 (202–330)	212 (140–260)
CSF-K^+^ (mmol/l)	2.90 (2.85–2.95)	2.90 (2.86–2.95)	2.86 (2.77–2.94)
CSF-Cl^−^ (mmol/l)	125 (122–127)	126 (125–127)	122 (121–122)***
CSF-Na^+^ (mmol/l)	147 (146–149)	147 (145–149)	148 (147–149)
CSF-tMg^2+^ (mmol/l)	1.14 (1.11–1.17)	1.15 (1.11–1.18)	1.12 (1.10–1.15)
CSF-tCa^2+^ (mmol/l)	1.17 (1.14–1.20)	1.19 (1.16–1.21)	1.15 (1.14–1.17)*
*N*_serum_	28	14	14
s-K^+^ (mmol/l)	4.17 (4.03–4.41)	4.13 (4.03–4.36)	4.22 (3.96–4.43)
s-Cl^−^ (mmol/l)	99.5 (98.5–100)	98.8 (97.3–100)	99.8 (99.2–101)*
s-Na^+^ (mmol/l)	140 (138–141)	140 (138–140)	140 (138–141)
s-tMg^2+^ (mmol/l)	0.83 (0.77–0.87)	0.85 (0.80–0.89)	0.82 (0.76–0.85)
s-tCa^2+^ (mmol/l)	2.34 (2.30–2.39)	2.34 (2.31–2.40)	2.34 (2.28–2.41)

Median (IQR). Fisher’s exact test was used to assess sex differences. Mann–Whitney *U* tests were employed for all other comparisons. Significance levels: *P < 0.05; **P < 0.01; ***P < 0.001.

All, healthy and symptomatic combined; IQR, interquartile range; Qalb, albumin quotient; GFAP, glial fibrillary acidic protein; NfL, neurofilament light chain; t-tau, total tau protein.

We found that [Cl^−^]_CSF_ (*P* < 0.0001) and [tCa^2+^]_CSF_ (*P* = 0.0050) were significantly lower in the symptomatic group while the differences in [Na^+^]_CSF_ (*P* = 0.50), [K^+^]_CSF_ (*P* = 0.11) and [tMg^2+^]_CSF_ (*P* = 0.057) were not statistically significant. [Cl^−^]_serum_ was significantly higher in the symptomatic group (*P* = 0.012). *P*-values for group differences were not adjusted for multiple comparisons.

Despite ostensible differences in age, GFAP, NfL, Cl^−^ and tCa^2+^ between the healthy and symptomatic subjects, we pooled all data for further analyses to increase statistical power.

### The major CSF electrolytes are each found in a narrow concentration range which is separate from their serum ranges

**Figure 2:**
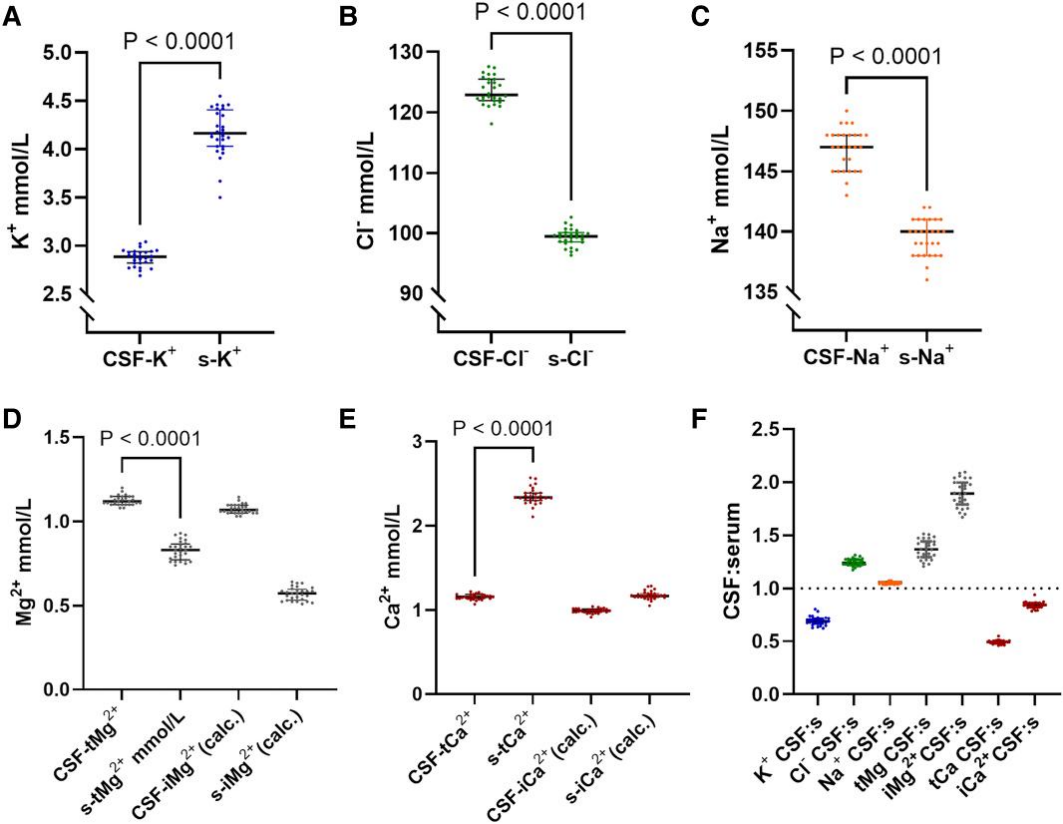
**The major CSF electrolytes are each found in a narrow concentration range which is separate from their serum ranges.** (**A–E**) Paired concentrations of K^+^, Cl^−^, Na^+^, Mg^2+^ and Ca^2+^ in CSF and serum (*N* = 28). (**F**) CSF:serum ratios for the measured ions, respectively (*N* = 28). Calculated ionized magnesium (iMg^2+^) and calcium (iCa^2+^) are included in **D–F** for illustrative purposes only; no statistical tests were applied to these derived estimates. Statistical comparisons are limited to paired CSF and serum total ion concentrations, tested using the Wilcoxon signed-rank test. Data are presented as median ± IQR, with each point representing one individual. CSF-, cerebrospinal fluid; s-, serum concentrations; tMg, total magnesium; iMg^2+^ (calc.), calculated ionized magnesium; tCa, total calcium; iCa^2+^ (calc.), calculated ionized calcium.

Using the pooled data, we characterized the normal CSF ion composition. Paired measurements showed that the major CSF electrolytes were found within narrow concentration ranges, distinctly separate from their serum counterparts (Fig. 2A–E). CVs were all <4% in CSF and <7% in serum (Supplementary Table 1), indicating precise regulation of all measured ion concentrations.

Concentrations of Cl^−^, Na^+^ and tMg^2+^ were higher in CSF than in serum, with ratios (CSF: serum) of 1.25, 1.05 and 1.37, respectively (Fig. 2B–D and F). Conversely, K^+^ and tCa^2+^ levels were lower in the CSF, with ratios of 0.70 and 0.50, respectively (Fig. 2A, E and F).

We estimated the free ionized fractions of Ca^2+^ and Mg^2+^, finding that the ionized Mg^2+^ ratio was higher (1.89) compared to the total Mg^2+^ ratio (1.37) (Fig. 2F). The ionized Ca^2+^ ratio (0.86) was closer to equity than the total Ca^2+^ ratio (0.50) (Fig. 2F).

### Factors correlating with CSF ion concentrations

Next, we investigated the potential influences on CSF ion concentrations, including serum ion levels, BBB integrity, age and circadian rhythmicity, using correlation analyses to examine the influence of these factors. The data were also dichotomized according to sex, for indications of hormonal influence on CSF ion concentrations.

#### K^+^, Cl^−^ and tMg^2+^ CSF concentrations did not significantly correlate with their respective serum concentrations

**Figure 3:**
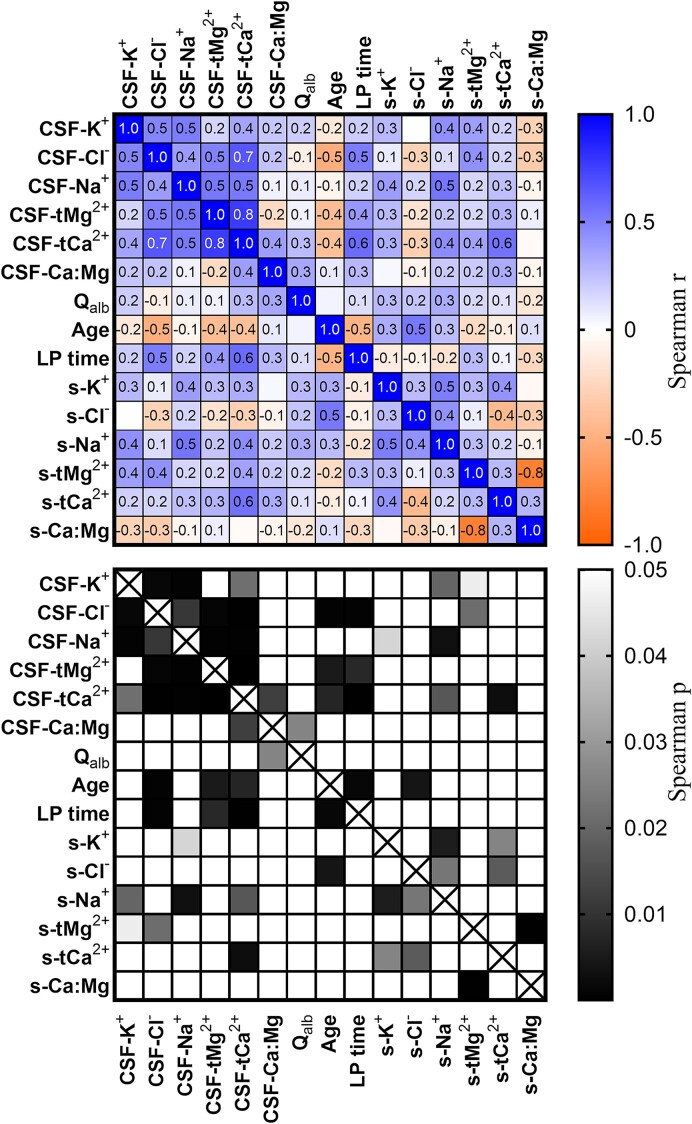
**Factors correlating with CSF ion concentrations.** Spearman *r* (top) and *p* (bottom) for respective ion in CSF (*N* = 42) and serum (*N* = 28), CSF-total calcium:total magnesium ratio, albumin quotient and age. *P*-values are not adjusted for multiple comparisons. CSF-, cerebrospinal fluid; s-, serum; tCa^2+^, total calcium; tMg^2+^, total magnesium; Ca:Mg: total calcium: total magnesium ratio; Q_alb_, albumin quotient; LP time, sampling (lumbar puncture) time.

To examine whether CSF and serum ion concentrations are separately controlled, we analysed correlations between the CSF ion concentrations and serum ion concentrations (Fig. 3; Supplementary Fig. 1). No significant correlations were found for [K^+^]_CSF_, [Cl^−^]_CSF_ or [tMg^2+^]_CSF_ (Fig. 3; Supplementary Fig. 1A, B and D) while [Na^+^]_CSF_ and [tCa^2+^]_CSF_ showed moderate correlations with their respective serum concentrations, significant only before adjusting for multiple comparisons (Fig. 3; Supplementary Fig. 1C and E).

Notably, both [Na^+^]_CSF_ and [Na^+^]_serum_ had positive Spearman correlation coefficients to all other ion concentrations. In fact, all CSF ion concentrations had positive correlation coefficients to all other ion concentrations in both fluids except to [Cl^−^]_serum_, which displayed correlations in both directions also to other serum concentrations. Moreover, [Cl^−^]_serum_ had a negative correlation coefficient to [Cl^−^]_CSF_.

These results suggest more precise homeostatic regulation of [K^+^]_CSF_, [Cl^−^]_CSF_ and [tMg^2+^]_CSF_ within the CNS, while [Na⁺]_CSF_ and [tCa²⁺]_CSF_ may reflect serum-driven regulatory mechanisms to a higher degree, implying distinct pathways for ion homeostasis in the CSF.

#### Blood–brain barrier integrity did not significantly correlate with CSF ion concentrations

As an indication of BBB integrity, we calculated Q_alb_ and measured CSF-PDGFRβ. CSF-PDGFRβ was only analysed in the 15 healthy samples not previously analysed, where it correlated significantly with Q_alb_ (*r* = 0.59; *P* = 0.022; *N* = 15). None of the analysed CSF-ion concentrations significantly correlated with Q_alb_ (Fig. 3; Supplementary Fig. 2), nor with PDGFRβ (data not shown). [Na⁺]_CSF_ and [tMg^2+^]_CSF_ even had weak correlation coefficients to Q_alb_ in opposite direction to expected if the barrier function of the BBB is of importance to sustain ion concentration gradients (Fig. 3; Supplementary Fig. 2C and D). These results indicate that BBB integrity in the physiological range has little, or no, role in limiting equilibration of CSF ion concentrations to blood.

#### Age and/or timing of lumbar puncture may impact CSF ion concentrations

Before adjusting for multiple comparisons, [Cl^−^]_CSF_, [tMg^2+^]_CSF_ and [tCa^2+^]_CSF_ were significantly correlated with sampling time of day, and inversely with age, although differences were small (Fig. 3; Supplementary Figs 3 and 4). Age and sampling time were inversely correlated (Fig. 3; Supplementary Fig. 4G), complicating interpretations. The only statistically significant age correlation for any measured serum ion was a positive [Cl^−^]_serum_ correlation (Fig. 3) effectively resulting in a moderately decreased [Cl^−^]_CSF:serum_ ratio with age (Spearman *r* = −0.65; *P* = 0.0001, data not shown).

To disentangle age correlations from sampling time, we added the CSF ion concentration values obtained from the samples collected in the morning after sleep (6–7 a.m.) in Forsberg *et al*.^18^ to the sampling time correlations (Supplementary Fig. 5). Regardless of sampling time, the samples from Forsberg *et al*.^18^ displayed a pattern of higher [Cl^−^]_CSF_, [tMg^2+^]_CSF_ and [tCa^2+^]_CSF_ than samples analysed in the other two batches, weakening sampling time correlations for these CSF ions to statistical non-significance. On the other hand, the positive time correlation coefficient for [K^+^]_CSF_ was strengthened, but only passed the threshold for significance before adjusting for multiple comparisons. Age correlations for [Cl^−^]_CSF_ (*r* = −0.52, *P* = 0.002), [tMg^2+^]_CSF_ (*r* = −0.51, *P* = 0.002) and [tCa^2+^]_CSF_ (*r* = −0.49, *P* = 0.004) were strengthened by adding the morning samples, all statistically significant after adjusting for multiple comparisons.

#### Men had marginally higher [tMg^2+^]_CSF_ and [tCa^2+^]_CSF_

**Figure 4:**
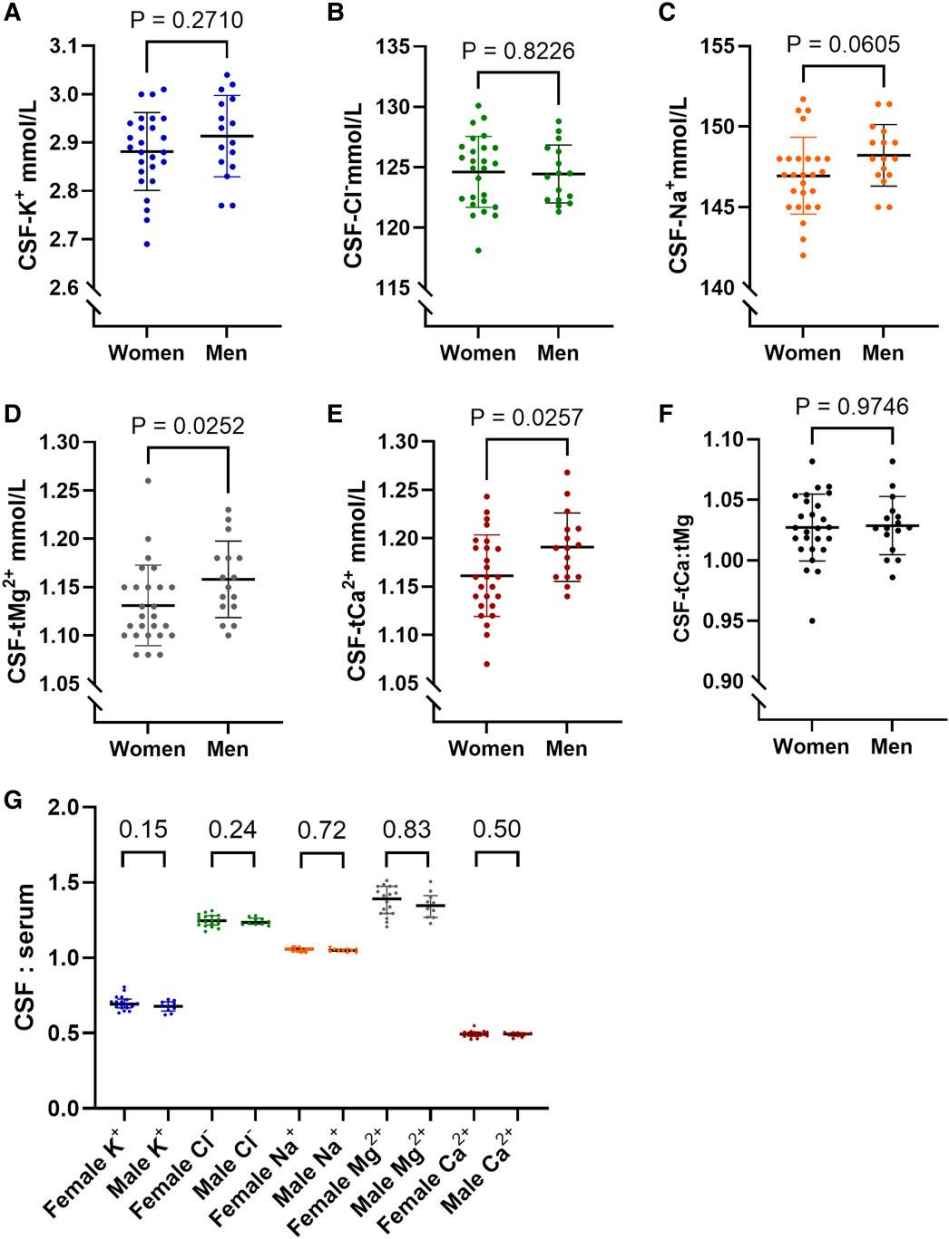
**Men had marginally higher [tMg^2+^]_CSF_ and [tCa^2+^]_CSF_.** (**A–E**) CSF concentrations of K^+^, Cl^−^, Na^+^, tMg^2+^ and tCa^2+^ in women (*N* = 26) and men (*N* = 16). (**F**) CSF tCa^2+^:tMg^2+^ ratios. (**G**) CSF:serum ratios of each ion in women (*N* = 18) and men (*N* = 10); each ion was analysed separately. Group differences in all panels (**A–G**) were tested using the Mann–Whitney *U* test for two independent groups. Unadjusted *P*-values are reported. Data are presented as median ± IQR, with each point representing one individual. CSF-, cerebrospinal fluid; tMg^2+^, total magnesium; tCa^2+^, total calcium.

Finally, we evaluated potential sex differences by dividing the samples into male and female groups. These groups were comparable in age, but men had substantially and significantly higher Q_alb_ (*P* = 0.010) (Table 2). Both [tMg^2+^]_CSF_ and [tCa^2+^]_CSF_ were slightly higher in men (Fig. 4D and E), statistically significant only before adjusting for multiple comparisons. No significant differences in the ratios of CSF to serum ion concentrations were observed between sexes in the subset of subjects with data from both fluids (Fig. 4G). Higher serum Na^+^ in men (*P* = 0.007) was the only ion concentration in any fluid that significantly differed between sexes after adjusting for multiple comparisons (Supplementary Fig. 6).

**Table 2:** Biomarker distributions between the sexes

	Women	Men
*N*_CSF_	26	16
Age (years)	25.5 (23–33)	25 (22–35)
Q_alb_	3.4 (2.4–4.0)	4.1 (3.5–5.1)*
GFAP (pg/ml)	314 (230–481)	315 (250–470)
NfL (pg/ml)	207 (119–280)	258 (174–350)
Tau (pg/ml)	252 (183–302)	228 (194–278)
CSF-K^+^ (mmol/l)	2.89 (2.84–2.94)	2.92 (2.85–2.99)
CSF-Cl^−^ (mmol/l)	125 (122–127)	124 (122–127)
CSF-Na^+^ (mmol/l)	147 (145–148)	148 (147–150)
CSF-Mg^2+^ (mmol/l)	1.12 (1.10–1.15)	1.16 (1.13–1.18)*
CSF-Ca^2+^ (mmol/l)	1.16 (1.13–1.19)	1.19 (1.16–1.21)*
*N*_serum_	18	10
s-K^+^ (mmol/l)	4.13 (4.00–4.23)	4.40 (4.14–4.45)*
s-Cl^−^ (mmol/l)	99.5 (98.0–100)	99.5 (99.0–100)
s-Na^+^ (mmol/l)	139 (138–140)	141 (140–141)*
s-Mg^2+^ (mmol/l)	0.82 (0.76–0.86)	0.84 (0.81–0.91)
s-Ca^2+^ (mmol/l)	2.32 (2.27–2.36)	2.39 (2.34–2.46)*

Median (IQR). Significance was determined with Mann-Whitney *U* tests with unadjusted significance levels presented: *0.01 < *P* < 0.05.

IQR, interquartile range; Q_alb_, albumin quotient; GFAP, glial fibrillary acidic protein; NfL, neurofilament light chain; t-tau, total tau proteins.

## Discussion

This study aims to guide future research by quantifying the normal human CSF composition of key ions for excitation and explore how the composition may be influenced by each CSF ion concentration, their paired serum concentrations, BBB permeability, age, timing of sample collection and sex. The findings show that each measured CSF ion concentration was distinctly separated, without overlap, from their respective serum concentration ranges, in concentrations which were either clearly higher (Cl^−^, Na^+^ and Mg^2+^) or lower (K^+^ and Ca^2+^) in the CSF, and only to a minor degree impacted by the analysed potentially confounding factors. These results demonstrate the precise regulation of CSF ion homeostasis and reinforces the long-known fact that concentration ranges in serum and CSF are separate (K^+^,^41-43^ Cl^−^ and Na^+^,^44^ Mg^2+4^ and Ca^2+3^) and that the CSF is not merely an ultrafiltrate of serum.^1,2^

Our results obtained from a young cohort of completely healthy individuals, and compared to a modern symptomatic control group, can be used as an estimate of normalized concentration ranges for CSF ion concentrations. These values show generally lower variability, but are otherwise in line with those previously obtained from ‘near to normal’ patients with varying and mostly unidentified or undisclosed, underlying conditions (Supplementary Table 2). These ranges can be compared to control groups of future clinical studies and to local normalized reference intervals for clinical use.

The low interindividual variability observed in our cohorts indicates favourable conditions for future studies to detect group differences in CSF ion concentrations. The correlation analysis showed a prominent pattern of positive correlations between the different ions in both CSF and serum, suggesting that hydration (dilution/concentration) influences absolute CSF ion concentrations. This led us to questioning whether the significant correlations for [Na^+^]_CSF_ and [tCa^2+^]_CSF_ with their serum ion concentrations represents a direct ionic equilibrium with serum. These correlations may rather reflect a lesser control of these ions within the CNS, resulting in higher susceptibility to dilution when serum hydration transfers to the CNS, while compensatory control of [K^+^]_CSF_, [tMg^2+^]_CSF_ and [Cl^−^]_CSF_ is more rigorous, in line with previous observations.^1,45^ Recently, thorough efforts have been made to account for interindividual variability in organic CSF biomarkers likely based on hydration by normalizing each individual’s biomarkers to reference proteins.^46,47^ If a similar approach would be evaluated for CSF ion concentrations, despite them displaying much more precise regulation, we would suggest normalizing to [Na^+^] or [Ca^2+^] in CSF (or even in serum/plasma).

Though the ionic composition of CSF is largely determined already during production in the choroid plexus, ionic regulation also inside the CNS seems active and efficient considering the further reduced [K^+^] in cisterna magna fluid^48^ and the stable K^+^ levels also when blood acutely enters the CSF.^49^ A proposed sensing and regulation of [K^+^] by the BBB is potentially facilitated by astrocytic uptake and siphoning,^50^ where K^+^ is shuttled between the neuropil and the BBB, aided by colocalized K^+^ and H_2_O channels at the astrocytic endfeet.^51^

Despite a more than 5-fold range in Q_alb_ in our cohort, no strong correlation was observed between Q_alb_ (or PDGFRβ) and CSF ion concentrations. This finding indicates that, under normal physiological conditions, even substantial variations in BBB permeability have little impact on ion homeostasis. This conclusion is consistent with the much greater transfer rates of ions across the BBB compared to albumin,^30^ meaning that ions pass the BBB in the direction of their concentration gradient relatively freely already under physiological permeability, but gradients are sustained through active transport in the opposite direction.

Our findings indicate that it may be important to consider subject age, sampling time and sex in future studies. We noted a significant (before adjusting for multiple comparisons) positive correlation between [K^+^]_CSF_ and sampling time (CSF-K^+^ increased about 1% in 8 h), supporting the previous conclusion of circadian regulation primarily of CSF-K^+^.^18^ The correlations between CSF ion concentrations and subject age were generally small ([Cl^−^]_CSF_, [tMg^2+^]_CSF_ and [tCa^2+^]_CSF_ decreased less than 1.5% of median over a decade), but clearly detectable. Also sex differences in CSF ion concentrations were small (about 3% of median for [tMg^2+^]_CSF_ and [tCa^2+^]_CSF_ and no more than 0.5–1%, if any, for the other measured CSF ions). Neither age, nor sex, effects were observed in a previous study on 155 children who underwent diagnostic lumbar puncture but had major neurological and inflammatory diseases ruled out.^52^

We propose a perspective where the unique ionic environment distinct from serum created by the precise regulation of K^+^, Cl^−^, Na^+^, Mg^2+^ and Ca^2+^ in the CSF, demonstrated in our study, likely serves to lower baseline neuronal excitability, facilitating sleep and protecting against overexcitation.

The lower K^+^ concentration in CSF compared to serum promotes hyperpolarization by maintaining a more negative K^+^ equilibrium potential, while the relatively larger elevation in Cl^−^ concentrations compared to Na^+^ further reduces excitability by shifting the Cl^−^ equilibrium potential more negatively relative to Na^+^. Higher Mg^2+53-55^ and lower Ca^2+56,57^ concentrations in CSF synergistically reduce neurotransmitter release by inhibiting Ca^2+^ influx through voltage-gated Ca^2+^ channels. Additionally, excitability is reduced by CSF from the relatively larger enrichment of iMg^2+^ compared to the deficit in iCa^2+^, by net increased charge screening effects on voltage-gated sodium channels,^58-60^ and from Mg^2+^ inhibiting excitatory NMDA receptors^61,62^ and stimulating inhibitory GABA_A_ receptors.^63^ These considerations emphasize the importance of using CSF ion concentrations, rather than serum ion concentrations, as a starting point when designing artificial CSF for *in vitro* brain studies. Furthermore, it should be noted that human CSF contains neuromodulators that promotes excitability, thus opposing the low excitability mediated by the ion concentrations, as shown by *in vitro* experiments comparing human CSF with artificial CSF containing matched ion concentrations.^33,64-66^

## Limitations

Despite adding symptomatic subjects without evidence of conditions that could affect normal CSF ion composition, the limited sample size and range of variables of the study may have underpowered it to clearly distinguish the exact effects of factors such as BBB permeability, age, timing of lumbar puncture or sex on the measured concentrations. Before inclusion, more than half of the subjects with medically unexplained symptoms were excluded, illustrating that identification of symptomatic controls by diagnosis codes alone does not guarantee a strictly physiological cohort. Even after our efforts to eliminate bias, ion concentrations and injury markers in this group still differed somewhat from the healthy group, indicating the importance of completely healthy individuals to obtain physiological references values.

A further limitation is that we did not directly measure the biologically active ionized fractions of Ca^2+^ or Mg^2+^ which seem to be hormonally regulated.^40^  ^,67^ This could limit our understanding of the full physiological impact of these ions on brain activity.

## Conclusions

Our findings demonstrate that CSF ion concentrations are precisely regulated, largely independent of serum levels and with low interindividual variation, reinforcing the concept of a highly controlled CNS environment. The distinct ion composition may help modulate neuronal excitability, supporting brain state transitions such as sleep–wake cycling. We identify hydration status as a potential confounding factor, particularly affecting Na^+^ and Ca^2+^ levels, and suggest that normalization to CSF-Na^+^ may improve the detection of pathological disruptions in CSF ion homeostasis. These insights lay a foundation for using CSF ion profiles as biomarkers for neurological disorders.

## Supplementary Material

fcaf201_Supplementary_Data

## Data Availability

The data sets used and/or analysed during the current study are available from the corresponding author on reasonable request.
