# Supplementary material for: Ion concentrations in CSF and serum are differentially and precisely regulated
Source: Brain Commun. 2025 May 24;7(3):fcaf201. doi: 10.1093/braincomms/fcaf201 (PMC12129886; doi:10.1093/braincomms/fcaf201)
Supplement: fcaf201_Supplementary_Data [file fcaf201_supplementary_data.pdf]

Supplementary Materials

Supplementary Table 1: Reference values for CSF electrolytes

**All**	**N**	**Median**	**IQR**	**Range**	**CV**	**95% CI**
**CSF-K^+** mmol/L	42	2.9	2.85-2.95	0.35	2.80%	2.9 - 2.9
**s-K^+** mmol/L	28	4.17	4.03-4.41	1.05	5.80%	4.1 - 4.4
**CSF-Cl^-** mmol/L	42	124.7	122-127	12	2.20%	123 - 126
**s-Cl^-** mmol/L	28	99.5	99-100	6.3	1.50%	99 - 100
**CSF-Na^+** mmol/L	42	147.3	146-149	9.7	1.50%	147 - 148
**s-Na^+** mmol/L	28	140	138-141	6	1.10%	139 - 140
**CSF-tMg** mmol/L	42	1.14	1.11-1.17	0.18	3.70%	1.1 - 1.2
**s-tMg** mmol/L	28	0.83	0.77-0.87	0.19	6.80%	0.8 - 0.9
**CSF-iMg^{2+}**(calc.)	42	1.08	1.06-1.12	0.17	3.70%	1.1 - 1.1
**s-iMg^{2+}**(calc.)	28	0.57	0.53-0.60	0.13	6.80%	0.6 - 0.6
**CSF-tCa** mmol/L	42	1.17	1.14-1.20	0.2	3.60%	1.2 - 1.2
**s-tCa** mmol/L	28	2.34	2.30-2.39	0.46	4.20%	2.3 - 2.4
**CSF-iCa^{2+}**(calc.)	42	1	0.98-1.03	0.17	3.60%	1 - 1
**s-iCa^{2+}**(calc.)	28	1.17	1.15-1.20	0.23	4.20%	1.2 - 1.2
**CSF-tCa:CSF-tMg**	42	1.03	1.01-1.05	0.13	2.50%	1 - 1
**s-tCa:s-tMg**	28	2.84	2.69-3.02	0.8	7.00%	2.7 - 3
**CSF-ions sum**	28	276	274-278	15	1.10%	275 - 277
**s-ions sum**	28	246	244-248	12	1.10%	244 - 248
**Q_{alb}**	41	3.73	2.65-4.43	5.92	35.40%	3 - 4.1

**Women**	**n**	**Median**	**IQR**	**Range**	**CV**	**95% CI**
**CSF-K^+** mmol/L	26	2.89	2.84-2.94	0.32	2.80%	2.9 - 2.9
**CSF-Cl^-** mmol/L	26	125.1	122-127	12	2.40%	122 - 127
**CSF-Na^+** mmol/L	26	147	145-148	9.7	1.60%	146 - 148
**CSF-tMg** mmol/L	26	1.12	1.10-1.15	0.18	3.70%	1.1 - 1.2
**CSF-tCa** mmol/L	26	1.16	1.13-1.19	0.17	3.60%	1.1 - 1.2
**CSF-tCa:CSF-tMg**	26	1.03	1.01-1.05	0.13	2.70%	1 - 1.1

**Men**	**n**	**Median**	**IQR**	**Range**	**CV**	**95% CI**
**CSF-K^+** mmol/L	16	2.92	2.85-2.99	0.27	2.90%	2.9 - 3
**CSF-Cl^-** mmol/L	16	124.4	122-127	7.5	1.90%	122 - 127
**CSF-Na^+** mmol/L	16	148	147-150	6.4	1.30%	147 - 150
**CSF-tMg** mmol/L	16	1.16	1.13-1.18	0.13	3.40%	1.1 - 1.2
**CSF-tCa** mmol/L	16	1.19	1.16-1.21	0.13	3.00%	1.2 - 1.2
**CSF-tCa:CSF-tMg**	16	1.03	1.01-1.04	0.1	2.30%	1 - 1

Abbreviations: n, number of samples; IQR, Inter Quartile Range; CV, Coefficient of Variation; CI, Confidence interval (of median); CSF-, Cerebrospinal Fluid; s-, serum; iCa^{2+} (calc.), calculated ionized calcium; iMg^{2+} (calc.), calculated ionized magnesium; CSF-tCa : CSF-tMg, total calcium : total magnesium ratios; ions sum, sums of intraindividually measured ion concentrations; Q_{alb}, albumin quotient.

Supplementary Table 2: Previously reported reference values for CSF electrolytes

Ion	Reference	N =	Method	Setting	Population	Mean	SD	Range
Na^+	*Liappis & Schneider 1984*[1]	155	FP	DW	*Children*	*132.3*	*17.6*	94-146
Na^+	Cooper et al. 1955[2]	20	SP	SA	Adults	141.2	6.0	
Na^+	Rotsch & Woratz 1960[3]	75	FP	All	All ages	143.5	5.7	
Na^+	Salminen & Luomanmäki 1962[4]*	13	FP	Other	Adults	145.4	3.4	
Na^+	Bradbury et al. 1963[5]	23	FP	DW	Adults	148.5	2.8	
Na^+	Montani & Perret 1964[6]	23	FP	DW	Adults	146.0	5.7	
Na^+	Sambrook 1974*	40	FP	DW	Adults	143.3	2.6	138-151
Na^+	Breyer & Quadbeck 1965[7]	116	FP	Other	Adults	148.0		142-154
Na^+	Kalin et al. 1975[8]	15	FP	CR	Adults	145.4	3.6	
Na^+	Kleine 1980[9]	50	FP	?	Adults	154.5	4.8	
Na^+	**Lyckenvik et al. 2025**	**42**	**ISE**	**HV+DW**	**Adults**	**147.4**	**2.3**	**142-152**
K^+	*Liappis & Schneider 1984*[1]	155	FP	DW	*Children*	*2.59*	*0.37*	1.82-3.59
K^+	Cooper et al. 1955[2]	20	SP	SA	Adults	2.96	0.45	
K^+	Rotsch & Woratz 1960[3]	76	FP	All	All ages	2.97	0.40	
K^+	Salminen & Luomanmäki 1962[4]*	13	FP	Other	Adults	2.82	0.15	
K^+	Bradbury et al. 1963[5]	23	FP	DW	Adults	2.88	0.15	
K^+	Montani & Perret 1964[6]	23	FP	DW	Adults	3.00	0.18	
K^+	Breyer & Quadbeck 1965[7]	30	FP	Other	Adults	2.83		2.68-3.00
K^+	Prill 1969*	50	FP	?	Adults	2.90		2.75-3.05
K^+	Breyer & Kanig 1970[10]	16	FP	Other	Adults	2.90	0.070	2.75-3.00
K^+	Sambrook 1974*	40	FP	DW	Adults	2.88	0.13	2.6-3.1
K^+	Kalin et al. 1975[8]	15	FP	CR	Adults	2.80	0.20	
K^+	Kleine 1980[9]	50	FP	?	Adults	2.90	0.25	
K^+	**Lyckenvik et al. 2025**	**42**	**ISE**	**HV+DW**	**Adults**	**2.89**	**0.082**	**2.69-3.04**
Cl^-	*Liappis & Schneider 1984*[1]	146	COL	DW	*Children*	*113.1*	*15.5*	79.8-126
Cl^-	Fremont-Smith 1931*	22	W&B	DW	Adults	124.1		119-128
Cl^-	Bradbury et al. 1963[5]	23	COL	DW	Adults	125.0	3.4	
Cl^-	Montani & Perret 1964[6]	23	FP	DW	Adults	119.4	4.8	
Cl^-	Sambrook 1974*	40	COL	DW	Adults	120.3	3.3	111-126
Cl^-	Breebart 1978[11]	139	FP	DW	Adults	125.0		119-132
Cl^-	Kleine 1980[9]	50	FP	?	Adults	122.4	4.5	113-131
Cl^-	**Lyckenvik et al. 2025**	**42**	**ISE**	**HV+DW**	**Adults**	**124.5**	**2.7**	**118-130**
Ca^{2+}	*Liappis & Schneider 1984*[1]	153	FL	DW	*Children*	*1.74*	*0.73*	0.78-3.29
Ca^{2+}	Hunter & Smith 1960[12]	38	?	DW	Adults	1.14	0.080	
Ca^{2+}	Rotsch & Woratz 1960[3]*	76	FP	All	All ages	1.23	0.018	

Ca^{2+}	Prill 1969[13]	50	?	?	Adults	1.21		1.10-1.43
Ca^{2+}	Kleine 1980[9]	50	?	?	Adults	1.23	0.28	
Ca^{2+}	**Lyckenvik et al. 2025**	**42**	**COL**	**HV+DW**	**Adults**	**1.17**	**0.042**	**1.07-1.27**
Mg^{2+}	*Liappis & Schneider 1984*[1]	125	XBR	DW	*Children*	*1.07*	*0.19*	0.71-1.56
Mg^{2+}	Hunter & Smith 1960[12]	38	FP	DW	Adults	1.12	0.045	
Mg^{2+}	Breyer & Quadbeck 1965[7]	54	FL	Other	Adults	1.20		1.14-1.28
Mg^{2+}	Breyer & Kanig 1970[10]	43	FL	Other	Adults	1.19	0.037	
Mg^{2+}	Apostol et al. 2010[14]*	10	COL	DW	Women	1.13		
Mg^{2+}	**Lyckenvik et al. 2025**	**42**	**COL**	**HV+DW**	**Adults**	**1.14**	**0.042**	**1.08-1.26**

References are from *Liappis & Schneider (1983)* with a few other prominent references added (marked with "*"). Abbreviations: FP, Flame Photometry; SP, Spectrophotometry; ISE, Ion Sensitive Electrode; COL, Colorimetry; W&B, Wilson and Ball method; XBR, Xylityl Blue Reaction; FL, Fluorimetry; DW, diagnostic workup – neurological conditions ruled out; SA, lumbar puncture during spinal anesthesia for elective surgery; All, unselected hospitalized patients analyzed within 24h of sampling; CR, sampling 24 hours after cardiac resuscitation; Other = other disease e.g. psychiatric conditions and vegetative disturbances; HV, Healthy Volunteers; Children, 1 month olds to 16 year olds; Women, adult women

1. Liappis N, Schneider A. [Reference values of sodium, potassium, chloride, calcium, inorganic phosphate and magnesium levels in the cerebrospinal fluid of children]. Klin Padiatr. 1984;196(6):370-4.
2. Cooper ES, Lechner E, Bellet S. Relation between serum and cerebrospinal fluid electrolytes under normal and abnormal conditions. Am J Med. 1955;18(4):613-21.
3. Rotzsch W, Woratz G. [Flame photometric determination of sodium, potassium and calcium in the cerebrospinal fluid]. Psychiatr Neurol Med Psychol (Leipz). 1960;12:65-7.
4. Salminen S, Luomanmaki K. Distribution of sodium and potassium in serum, cerebrospinal fluid, and serum ultra-filtrate in some diseases. Scand J Clin Lab Invest. 1962;14:425-9.
5. Bradbury MW, Stubbs J, Hughes IE, et al. The distribution of potassium, sodium, chloride and urea between lumbar cerebrospinal fluid and blood serum in human subjects. Clin Sci. 1963;25:97-105.
6. Montani S, Perret C. [Lactic acidosis of the cerebrospinal fluid in bacterial meningitis]. Schweiz Med Wochenschr. 1964;94:1552-7.
7. Breyer U, Quadbeck G. Der Gehalt des Liquor cerebrospinalis an Magnesium und anderen Kationen bei zentralnervösen Erkrankungen. Deutsche Zeitschrift für Nervenheilkunde. 1965;187(6):595-607.
8. Kalin EM, Tweed WA, Lee J, et al. Cerebrospinal-fluid acid-base and electrolyte changes resulting from cerebral anoxia in man. N Engl J Med. 1975;293(20):1013-6.
9. Kleine TO. Neue Labormethoden für die Liquordiagnostik. Stuttgart ; New York: Thieme; 1980.
10. Breyer U, Kanig K. Cerebrospinal fluid electrolyte disturbances in neurological disorders. Neurology. 1970;20(3):247-.
11. Breebaart K, Becker H, Jongebloed FA. Investigation of Reference Values of Components of Cerebrospinal Fluid. Clinical Chemistry and Laboratory Medicine (CCLM). 1978;16(10):561-6.
12. Hunter G, Smith HV. Calcium and magnesium in human cerebrospinal fluid. Nature. 1960;186:161-2.
13. Funktionen n, Prill A. Die Bedeutung des K+/Ca++-Quotienten sowie der isolierten Kalium-Erhöhung im Liquor cerebrospinalis für die Beurteilung zentralnervöser Funktionen. Deutsche Medizinische Wochenschrift. 1969;94:1743 - 9.
14. Apostol A, Apostol R, Ali E, et al. Cerebral spinal fluid and serum ionized magnesium and calcium levels in preeclamptic women during administration of magnesium sulfate. Fertil Steril. 2010;94(1):276-82.

Supplementary Figure 1. K^+, Cl^-, and $tMg2^+$ CSF concentrations did not correlate with their serum concentrations.

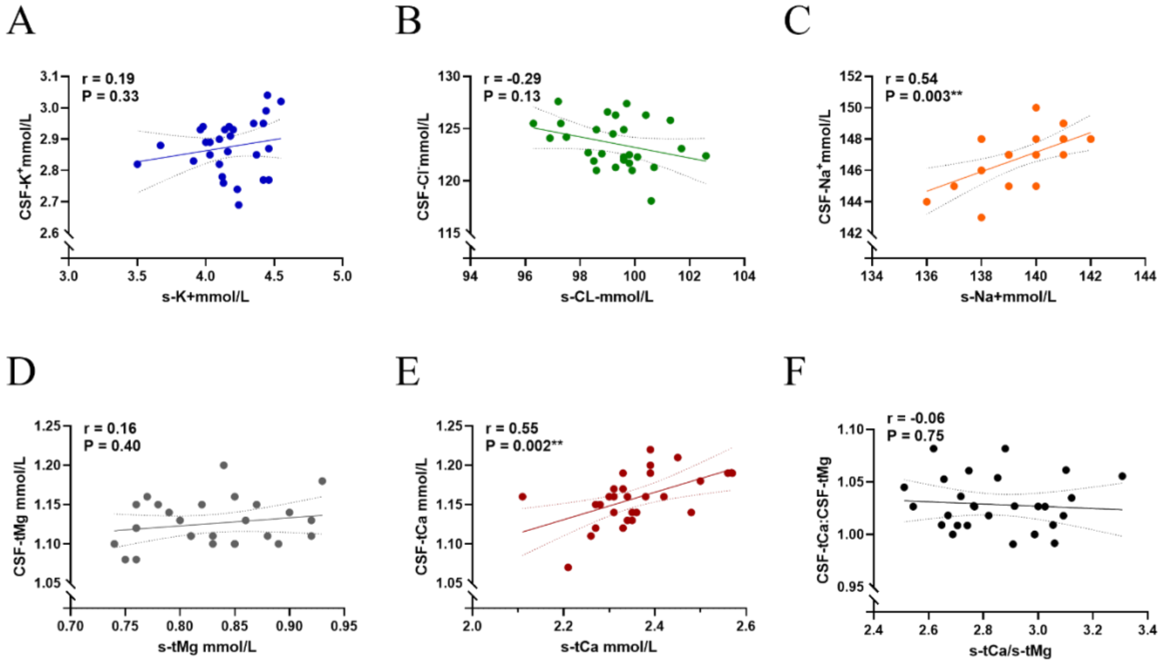

A-E: Scatter plots of paired serum and CSF concentrations for respective ion. F: Scatter plot for total calcium : total magnesium ratios in serum vs CSF. Each point represents measurements from one individual (N = 28). A linear regression line with 95% confidence interval is superimposed on each plot for visualization. The reported *r* and *p* values represent the Spearman correlation between the X- and Y-axes. Unadjusted p-values are shown.

Supplementary Figure 2. Blood-brain barrier integrity did not correlate with CSF ion concentrations.

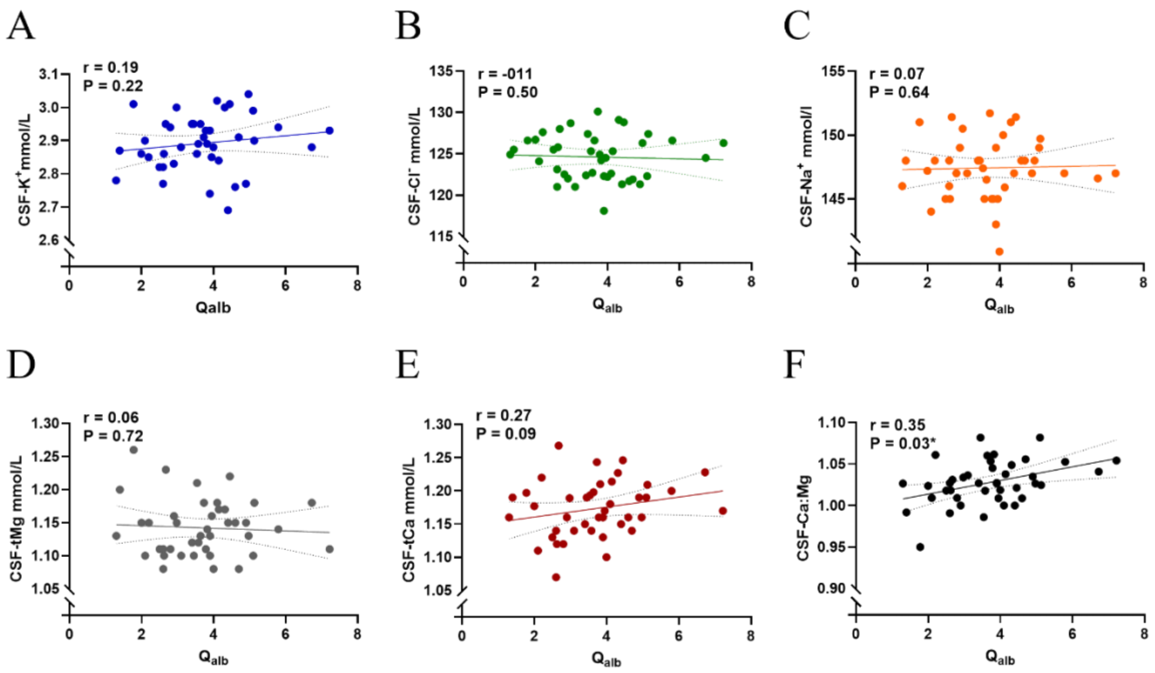

A-E: Scatter plots for respective ion plotted against albumin quotient. F. Scatter plot for CSF-total calcium : total magnesium ratio plotted against albumin quotient. Each point represents measurements from one individual (N = 41). A linear regression line with 95% confidence interval is superimposed on each plot for visualization. The reported *r* and *p* values represent the Spearman correlation between the X- and Y-axes. Unadjusted p-values are shown.

Supplementary Figure 3. Age may impact CSF ion concentrations.

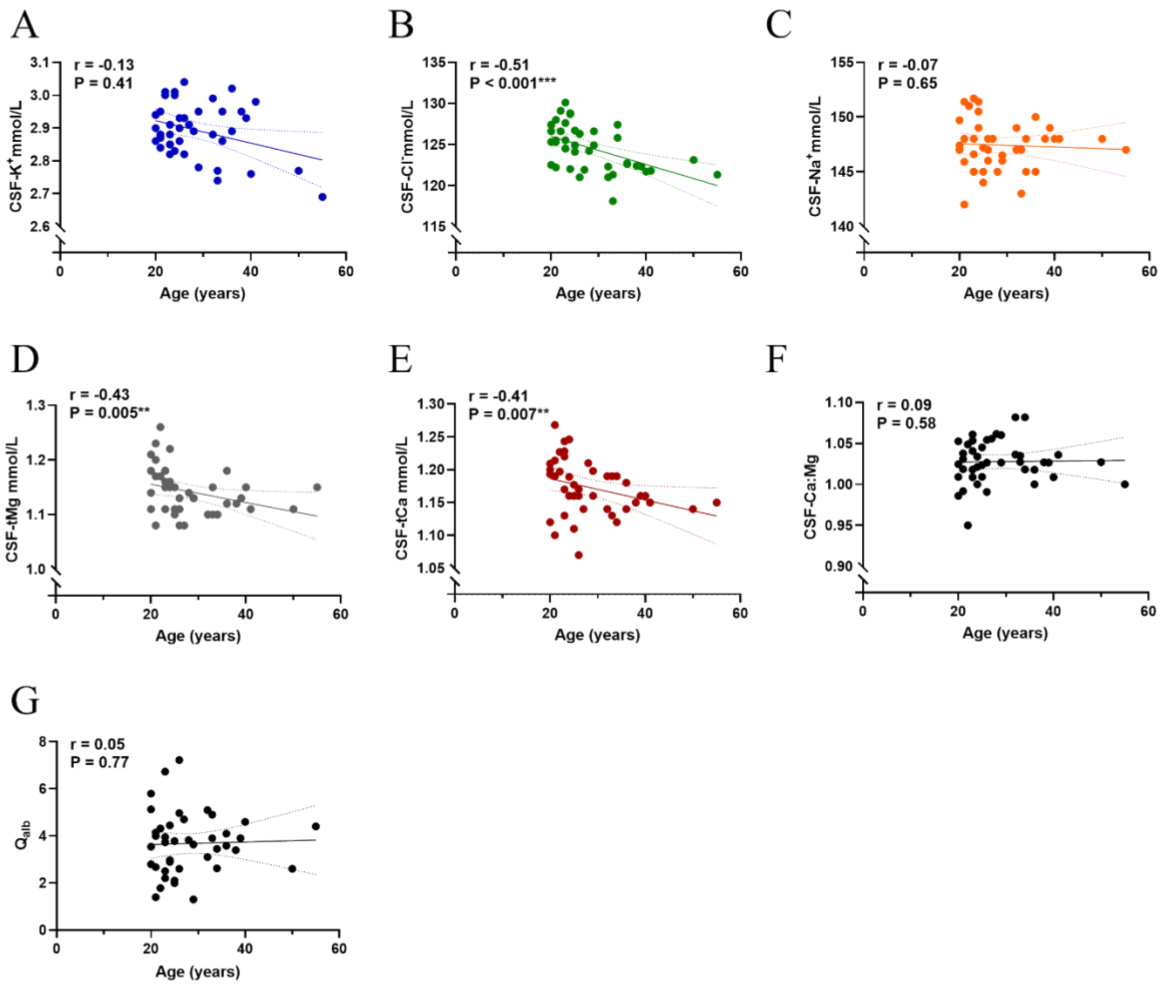

A-E: Scatter plots for respective ion plotted against age. F: Scatter plot for CSF-total calcium : total magnesium ratio plotted against age. G: Scatter plot of albumin quotient plotted against age. Each point represents measurements from one individual (N = 42). A linear regression line with 95% confidence interval is superimposed on each plot for visualization. The reported *r* and *p* values represent the Spearman correlation between the X- and Y-axes. Unadjusted p-values are shown.

Supplementary Figure 4. Timing of lumbar puncture may impact CSF ion concentrations.

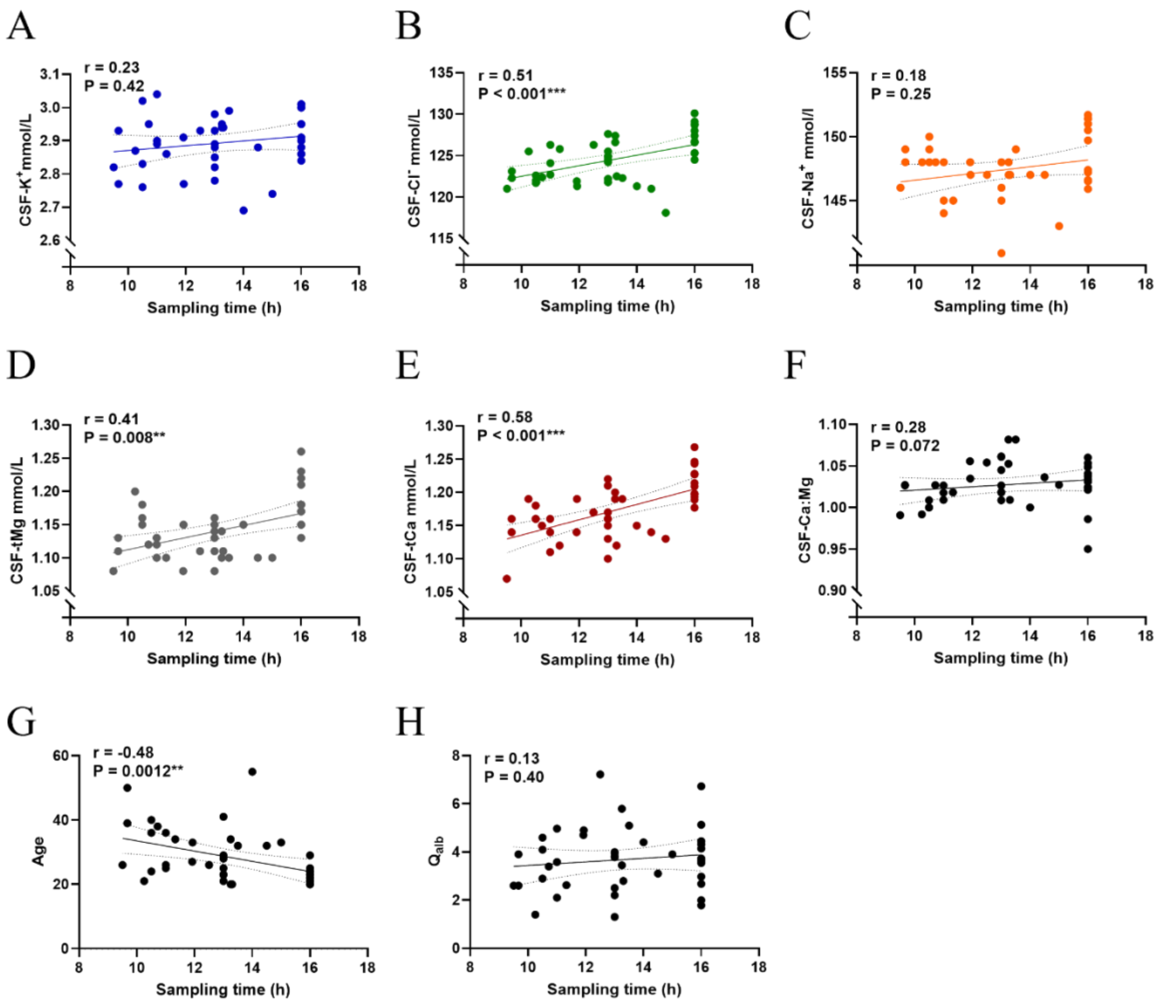

A-E: Scatter plots for respective ion plotted against sampling time. F. Scatter plot for CSF-total calcium : total magnesium ratio plotted against sampling time. G-H: Scatter plots for age and Q_{alb} plotted against sampling time. Each point represents measurements from one individual (N = 42). A linear regression line with 95% confidence interval is superimposed on each plot for visualization. The reported *r* and *p* values represent the Spearman correlation between the X- and Y-axes. Unadjusted p-values are shown.

Supplementary Figure 5. Timing of lumbar puncture may impact CSF ion concentrations, morning samples from *Forsberg et al. (2021)* added.

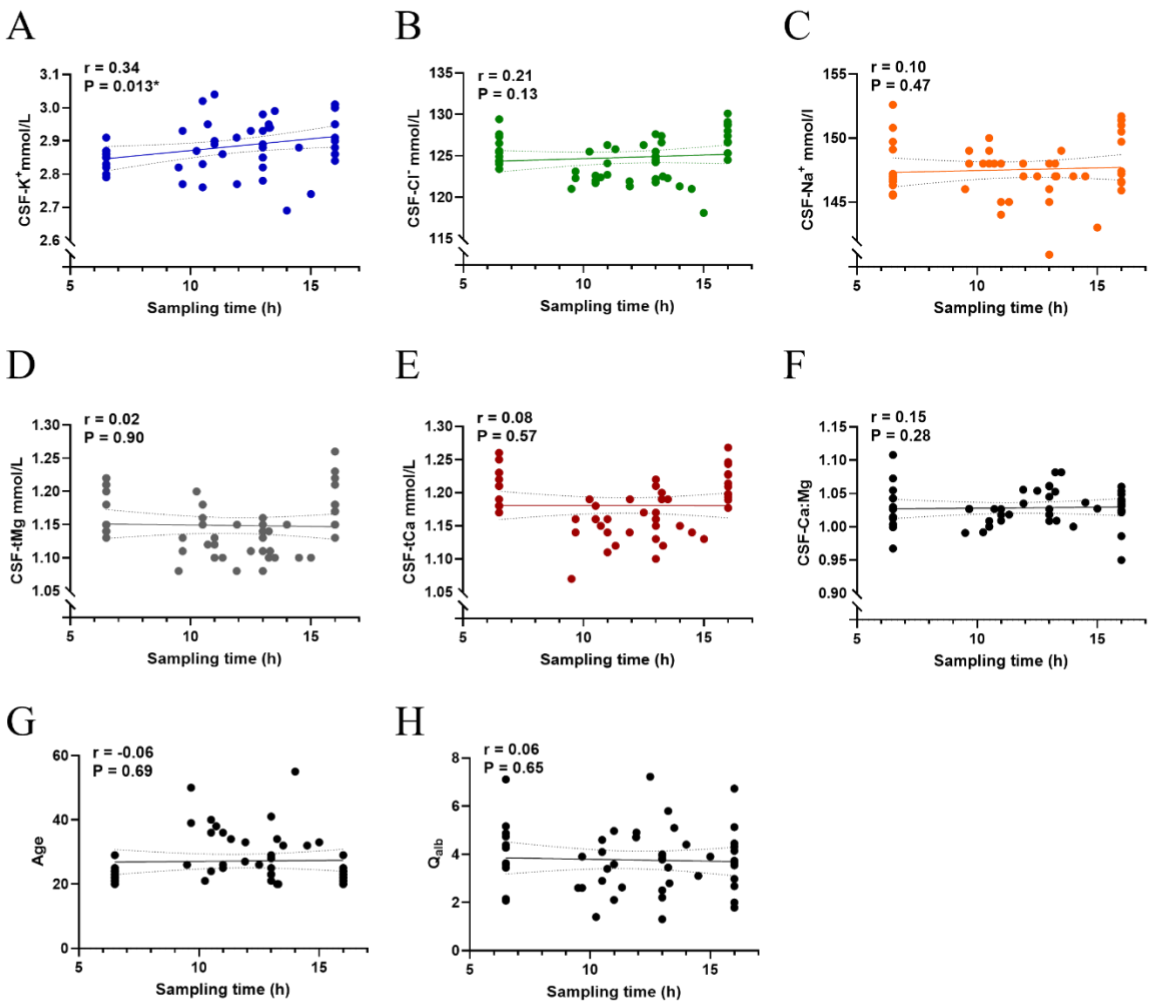

A-E: Scatter plots for respective ion plotted against sampling time. F. Scatter plot for CSF-total calcium : total magnesium ratio plotted against sampling time. G-H: Scatter plots for age and Q_{alb} plotted against sampling time. Each point represents measurements from one individual (N = 54). For 12 individuals, samples were collected at both 06:30 and 16:00 (at least four weeks between samples), yielding paired data points. A linear regression line with 95% confidence interval is superimposed on each plot for visualization. The reported *r* and *p* values represent the Spearman correlation between the X- and Y-axes. Unadjusted p-values are shown.

Supplementary Figure 6. Marginally higher serum ion concentrations in men.

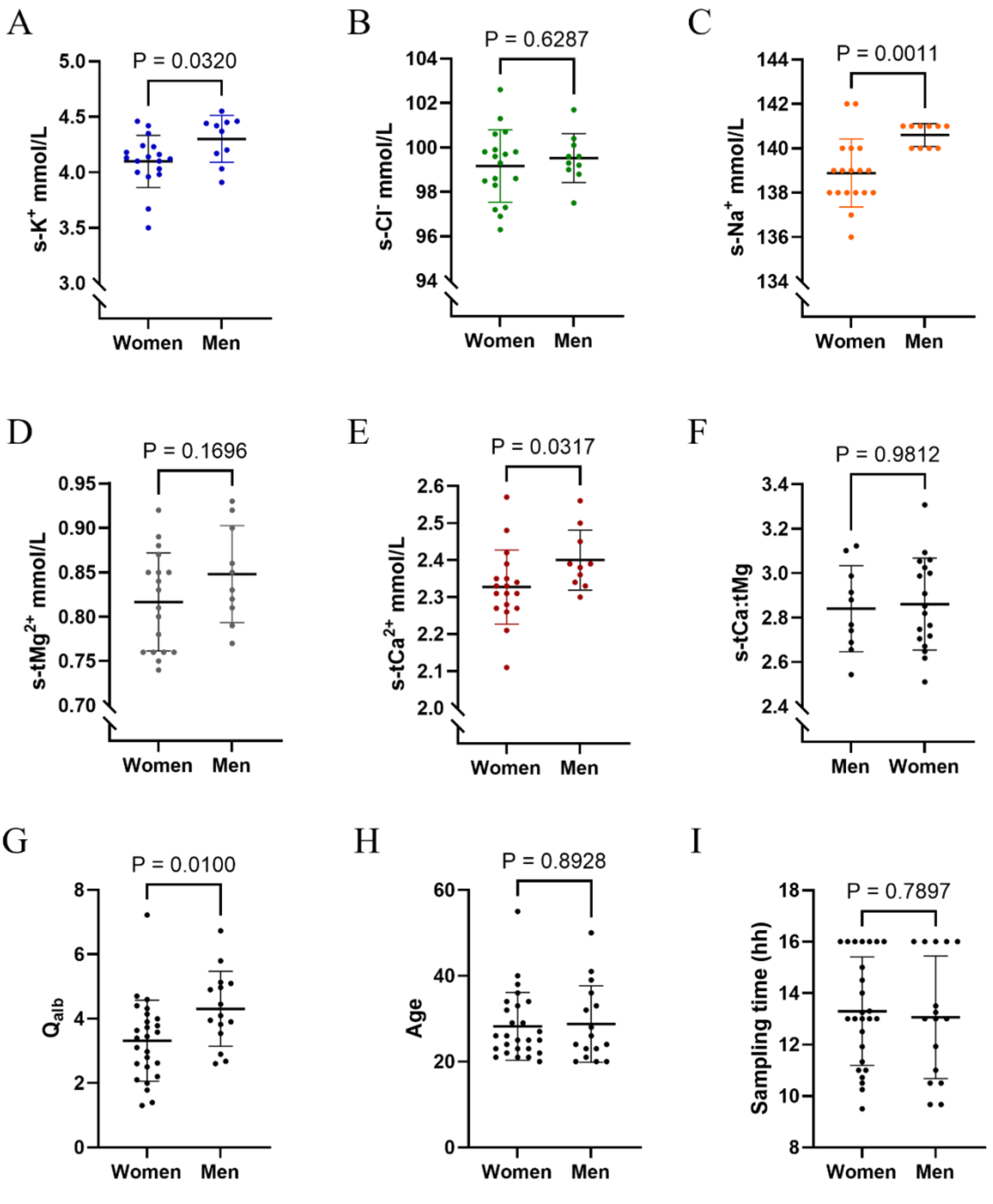

A-E: Serum concentrations of respective ion for women (N = 18) and men (N = 10). F: Total calcium : total magnesium ratios in serum for women and men. G: albumin quotient for women and men. H: Age (years) for women and men. I: Sampling time for women and men. Abbreviations: s-, serum concentrations; tMg^{2+}, total magnesium; tCa^{2+}, total calcium; Q_{alb}: albumin quotient. Data are shown as median ± IQR where each point represents a measurement from one individual. Significance was tested using Mann-Whitney test of unpaired differences between groups. Unadjusted p-values are shown.
